# Once-daily fluticasone furoate/vilanterol versus twice daily combination therapies in asthma–mixed treatment comparisons of clinical efficacy

**DOI:** 10.1186/s40733-015-0016-0

**Published:** 2016-02-08

**Authors:** Henrik Svedsater, Gillian Stynes, Jaro Wex, Lucy Frith, David Leather, Emanuela Castelnuovo, Michelle Detry, Scott Berry

**Affiliations:** 1 Value Evidence and Outcomes, GlaxoSmithKline, Stockley Park West, Blg 10, 1-3 Iron Bridge Road, Uxbridge, Middlesex UB11 1BT UK; 2 Respiratory Medicines Development Centre, GlaxoSmithKline, Stockley Park, UK; 3 Health Investment Evidence (Formerly), Global Health Outcomes, GlaxoSmithKline, Stockley Park, UK; 4 Berry Consultants LLC, Austin, TX USA

**Keywords:** Asthma, Fluticasone furoate, ICS/LABA, Mixed treatment comparison, Network meta-analysis, Vilanterol

## Abstract

**Background:**

Fluticasone furoate (FF)/vilanterol (VI) is a once-daily inhaled corticosteroid (ICS)/long-acting beta_2_ agonist (LABA) combination. FF/VI, 92/22mcg and 184/22mcg, are approved in Europe as maintenance therapy in persistent asthma. We report data from mixed treatment comparisons (MTC) of once-daily FF/VI against established twice-daily ICS/LABA combination therapies on clinical efficacy outcomes.

**Methods:**

Data from 31 parallel-group randomised controlled trials (RCTs) of ICS/LABA, of ≥8 weeks’ duration in patients aged ≥12 years with asthma, identified by systematic review, were analysed using covariate-adjusted Bayesian hierarchical models for four efficacy outcomes (primary analysis). Lung function, assessed by change from baseline morning peak expiratory flow (PEF) (*n* = 18 studies) and forced expiratory volume in 1 s (FEV_1_) (*n* = 28), was the outcome of primary interest. Secondary objectives were assessment of relative efficacy in terms of exacerbation rates (*n* = 6) and health status (*n* = 7). Overall, 24 different treatment arms were included in the MTC; we report findings comparing FF/VI (92/22mcg and 184/22mcg) with fluticasone propionate/salmeterol (FP/SAL) (250/50mcg and 500/50mcg) and budesonide/formoterol (BUD/FORM) (320/9mcg and 640/18mcg).

**Results:**

For PEF (margin = 12 l/min), FF/VI 92/22mcg demonstrated ≥94 % probability and FF/VI 184/22mcg >99 % probability of non-inferiority to corresponding doses of both FP/SAL and BUD/FORM. For FEV_1_ (margin = 100 ml), FF/VI demonstrated ≥98 % (92/22mcg) and >99 % (184/22mcg) probability of non-inferiority to both FP/SAL and BUD/FORM. Findings for exacerbations were inconclusive due to lack of data: FF/VI 92/22mcg demonstrated 74 % and 82 % probability of non-inferiority (margin = 10 %) to FP/SAL 250/50mcg and BUD/FORM 320/9mcg, respectively. For Asthma Quality of Life Questionnaire (AQLQ) score, FF/VI 92/22mcg demonstrated >99 % and 90 % probability of non-inferiority (margin = 0.25) to FP/SAL 250/50mcg and BUD/FORM 320/9mcg. Data were unavailable to assess non-inferiority of FF/VI 184/22mcg on exacerbations or AQLQ.

**Conclusions:**

Both strengths of once-daily FF/VI in asthma were comparable with corresponding doses of twice-daily FP/SAL and BUD/FORM in terms of lung function in this MTC analysis. FF/VI 92/22mcg was comparable with FP/SAL and BUD/FORM on AQLQ, but exacerbation results were inconclusive. Model limitations include disconnected treatment networks and variability across studies. Our data support previous RCT findings suggesting that the efficacy of once-daily FF/VI in improving lung function and health status in asthma is comparable with twice-daily ICS/LABAs.

**Electronic supplementary material:**

The online version of this article (doi:10.1186/s40733-015-0016-0) contains supplementary material, which is available to authorized users.

## Background

Fluticasone furoate (FF)/vilanterol (VI) is an inhaled corticosteroid (ICS)/long-acting beta_2_ agonist (LABA) combination approved in 2013 in Europe and Japan for the treatment of asthma. Both FF and VI have been shown to display 24-h activity in pre-clinical studies [1, 2], and a single daily dose of FF/VI has been shown to produce long-lasting improvement in lung function in patients with asthma [3]. Once-daily FF/VI, at strengths of 92/22 mcg and 184/22 mcg, has been shown to be well tolerated, with no safety profile findings of significant clinical concern, in a 12-month randomised controlled trial in patients aged ≥12 years with persistent moderate-severe asthma [4]. FF/VI therefore represents the first once-daily combination therapy approved for use in asthma.

The efficacy of FF/VI in asthma has been assessed in several randomised controlled trials (RCTs). The effects of once daily FF/VI 92/22 mcg (delivered dose; nominal dose is 100/25 mcg) on lung function and patient-reported health status have been compared in RCTs with FF 100 mcg [3, 5], and once-daily FF/VI 184/22 mcg (delivered dose; nominal dose is 200/25 mcg) has been compared with once-daily FF 200 mcg alone and twice-daily FP 500 mcg [6]. Once-daily FF/VI 92/22 mcg has also been compared in a head-to-head study with twice daily FP/SAL 250/50 mcg [7]. One study of the efficacy in reducing exacerbation rate of FF/VI 92/22 mcg compared with FF monotherapy has been reported [5]. However, the efficacy of once-daily FF/VI has not, as of the time of writing, been directly compared in any RCT with that of twice-daily combination therapies other than FP/SAL; some of which, such as budesonide (BUD)/formoterol (FORM), are widely used in the clinical management of asthma.

We sought to evaluate the relative therapeutic efficacy of FF/VI in asthma by making maximal use of the body of RCT data available. Indirect treatment comparisons, such as the mixed treatment comparison (MTC), provide a means of estimating the relative efficacy of treatments that have not been directly compared in an RCT, and broaden the evidence base for those treatments which have already been compared in head-to-head studies. We applied the MTC approach to compare the treatment efficacy of once-daily FF/VI 92/22 mcg and 184/22 mcg with corresponding strengths of alternative licensed twice-daily ICS/LABA combination therapies on four clinically-relevant outcomes. For each outcome, we combined data from separate RCTs, all of which were identified through a systematic literature review and involved at least one ICS/LABA comparator, in Bayesian, hierarchical models. These models were used to make inferences about relative treatment efficacy within the combined ICS/LABA therapy class and derive probabilities of non-inferiority and superiority for four clinically relevant outcomes: peak expiratory flow (PEF), forced expiratory volume in 1 s (FEV_1_), moderate/severe exacerbation rate, and asthma quality of life questionnaire (AQLQ/AQLQ(S) Total score). The primary focus of the analyses was on the non-inferiority results.

## Methods

### Systematic literature review

A systematic literature review was conducted to identify Phase III and Phase IV parallel-group RCTs of any ICS/LABA maintenance therapies vs any drug, placebo or standard of care comparator, of >8 weeks duration, in patients aged ≥12 years with asthma who were receiving ICS or ICS/LABA maintenance therapy at randomisation. Studies that required patients to be uncontrolled/symptomatic at baseline were eligible for inclusion in the MTC. Studies that included patients who were controlled/asymptomatic at baseline, predominantly recruited stable patients (explicitly defined or suggested by baseline averages of key parameters) or required patients to have a history of stable asthma, or did not report control or symptom status at randomisation were excluded. Additionally, studies containing ≥1 flexible dosing or dose-ranging arm were excluded.

A secondary MTC analysis, the ‘ICS-only subset analysis’, was conducted including only the subset of studies requiring patients to be treated with ICS only at baseline, i.e. LABA use was not permitted at randomisation. This approach identified studies in patient populations likely to be eligible for treatment with FF/VI in accordance with its European licence.

FF/VI trials were identified internally, and additional studies were identified through the systematic searching of clinical publication databases and clinical trial registers. Additionally, references in retrieved articles and relevant systematic reviews were checked for further studies that might fulfil the inclusion criteria. No date limits were applied to the searches.

### Outcome assessment

The outcomes assessed were change from baseline in lung function outcomes: PEF and FEV_1_, annual rate of moderate/severe exacerbations, and change from baseline in AQLQ health status questionnaire Total score. Moderate/severe exacerbations were defined according to the American Thoracic Society/European Respiratory Society Task Force recommendation [8] as deteriorations of asthma requiring the use of systemic/oral corticosteroids for ≥3 days, or emergency department visit or in-patient hospitalisation due to asthma requiring the use of systemic corticosteroids.

The outcome of primary interest, effect of treatment on lung function, was studied through the assessment of two measures of airflow limitation: PEF and FEV_1._ These measures are typically well correlated with each other [9].

For each outcome, studies identified through the systematic literature review were included in the MTC if they reported the precise outcome or sufficient calculable information in a suitable format. For PEF, to minimise study-to-study variability, studies were included only if they reported mean changes from baseline averaged over the entire duration of the trial. For exacerbations, studies were included in the MTC if they reported either the rate or number of moderate/severe exacerbations as defined above. Studies that allowed changes in symptoms, rescue use or lung function to be classed as exacerbations were excluded, as were those that withdrew patients once they experienced an exacerbation. With a single exception of arms consisting of steroid-naive patients in one study [10], all treatment arms from included studies were incorporated into the MTC.

The selection of margins that were used in the probabilistic assessment of non-inferiority for each outcome was informed by the margins specified in the design of previously reported comparative studies. For PEF, non-inferiority margins of 12 l/min and 15 l/min were selected on the basis of published guidelines [11]. For exacerbations, event rate ratios of 0.1 and 0.2 were used as non-inferiority margins on the basis of previous comparative studies [12]. For FEV_1_ (75 ml, 100 ml and 125 ml) and AQLQ (0.25 and 0.5), the non-inferiority margins used were based upon the accepted minimal clinically important difference (MCID) [13, 14].

### Modelling strategy

The MTC modelling approach used in this analysis employed a Bayesian, hierarchical methodology to estimate relative treatment effects, accounting for variability across studies by parameterising the study effect. The Bayesian approach [15–17] was decided upon prior to the commencement of the systematic literature review. This methodology enables the comparison of the effects of treatments that have never been directly compared in the same clinical trial, and is compatible with inference from weak or disconnected treatment networks [18].

For each outcome, a hierarchical random effects model was constructed in which the effect of each included study $\alpha$ was modelled with a distribution *α*_*S*_ ~ *N*(*μ*, *τ* ^2^). The two parameters *μ* and *τ* were then modelled with second-level hyperpriors and a posterior distribution created. Treatment effects, with independent prior distributions, were modelled separately as a single parameter, thus enabling probabilistic treatment comparisons to be derived together with credible intervals (CrI) for the differences in effect sizes.

For PEF, FEV_1_ and AQLQ Total score, the continuous change from baseline outcomes was modelled using Normal distributions. The mean treatment effect was modelled with the following distribution: *Y* ~ *N*(*α*_*s*_ + *θ*_*t*_ + *βZ* , *σ* ^2^), with the non-informative prior *σ*_*α*_^2^ ~ *Inverse* − *Gamma*(0.001, 0.001) and hyperpriors *μ*_*s*_ ~ *N*(0, 10^2^) and *τ* ^2^ ~ *Inverse* − *Gamma*(0.001, 0.001). The parameters *α* and *θ* represent, respectively, the studies included in the analysis, and the treatment regimen effects. The *Z*’s represent the covariates and the *β*’s represent the coefficients (i.e. the covariate effects). Each treatment effect is modelled independently with the flat prior distribution *N*(0, 100^2^).

For exacerbations, the yearly rate was modelled using a Poisson distribution, as follows: Exac ~ Poisson(Rate*person-years), in which *log(Rate) = α*_*s*_ + *θ*_*t*_ + *βZ*. Priors and model parameters for the study and treatment effects were defined as for the other three outcomes; treatment regimen effects are represented by *θ*. Each treatment effect is modelled independently with the flat prior distribution *N*(0, 5^2^). The placebo arms are designated as *t* = 0 and are used as the baseline treatment. The original analysis plan was to use a standard deviation of 100 for the treatment effects. This represents a very flat, non-informative prior distribution. However, the outcome of one trial arm with 0 events makes for a non-identifiable outcome which, combined with the very flat prior, creates numerical stability issues. Therefore, a vague prior of 5 is selected for the treatment arms, creating stable estimates and more interpretable model results.

Rate was defined as number of exacerbations divided by person-years of follow-up. Person-years of follow-up were computed directly if both the rate and number of events were available or were estimated if neither were available. When estimated, subjects not lost to follow-up were assumed to have had complete (100 %) follow-up. Subjects lost to follow-up were assumed to have 50 % of the possible follow-up. When the number of events was not reported, the rate and estimated person-years of follow-up were used to estimate the number of moderate/severe exacerbation events.

The following covariates were included in the full covariate model for all outcomes: study duration; age at baseline; gender; baseline mean FEV_1_. The continuous covariates–age, gender and baseline mean FEV_1_–were normalised. Specifically, age was normalised by subtracting 40 years of age; the resulting covariate is “Age–40”. Gender is represented as the percentage of males in a treatment arm. Gender was normalised by subtracting 40 % from the treatment arm population of males, thus the covariate is “%Male–40”. Baseline FEV_1_ is normalised by subtracting 2.4; the resulting covariate is “FEV_1_–2.4”. The single categorical variable, length of study, had a designated reference group of studies 40–60 weeks in length, i.e. those that were approximately 1 year in length.

In treatment arms with missing covariate information, mean values were imputed across all treatment arms. Attempts were made to fit full covariate models for all outcomes of interest, but this could not be achieved for exacerbations as data limitations meant the full covariate model did not converge. As such, exacerbation data are reported only for the time-covariate model, in which only study duration was modelled as a covariate. For the other three outcomes, findings from the full covariate model are reported.

Model fit was assessed by evaluating the difference between the model-estimated values and observed values, divided by the estimated standard deviation (SD).

All analyses were carried out using standard Markov Chain Monte Carlo methodology, utilising adaptive Metropolis-Hastings steps where applicable [19], and were performed using custom software written in ANSI-standard Fortran (Berry Consultants LLC, Austin, TX). The software used was independently validated with duplicate code written in R (R Development Core Team, Vienna, Austria).

### Sensitivity analysis

Sensitivity analyses, in which studies that were excluded under the primary analysis criteria were added to the model, were performed for the PEF and exacerbation rates outcomes of interest, using the same modelling approach as in the main analyses. For the outcome of change from baseline PEF, the sensitivity analysis included an additional nine studies that were excluded from the main analysis because they did not report change in PEF from baseline to study end. The PEF sensitivity analysis network was thus constructed using data from 27 studies. For exacerbations, one of the sensitivity analyses included an additional six studies that were excluded from the main analysis because they withdrew patients and discontinued their follow-up after they reported an exacerbation, for a sensitivity analysis network consisting of data from 12 studies.

In separate sensitivity analyses performed for the exacerbation rate network, the data for the original six studies were re-analysed using alternate versions of the person-years calculation for those cases where person-years were estimated. Two such analyses were conducted, using an assumed follow-up time for patients lost to follow-up of 25 % or 75 %, respectively, rather than 50 % as in the main analysis.

### Assessment of alternative modelling approaches

Two post-hoc validation analyses were performed on the same dataset using alternative methods in order to evaluate the extent to which outcomes were susceptible to the primary model chosen: a frequentist analysis using a random effects model with fixed study and treatment effects, reporting *p*-values was performed on all four outcomes of interest using R (lme4 package); and a pairwise contrast analysis using a Bayesian random effects model was performed on the FEV_1_ data only using geMTC software [20] running WinBUGS [21]. As geMTC did not enable automated analyses of rates with Poisson distributions, exacerbation rates were approximated as continuous variables, comparing rate differences rather than rate ratios. In the validation analyses, point estimates with confidence or credible intervals were calculated, with the frequentist analyses also reporting *p*-values.

## Results

### Study selection

**Fig. 1:**
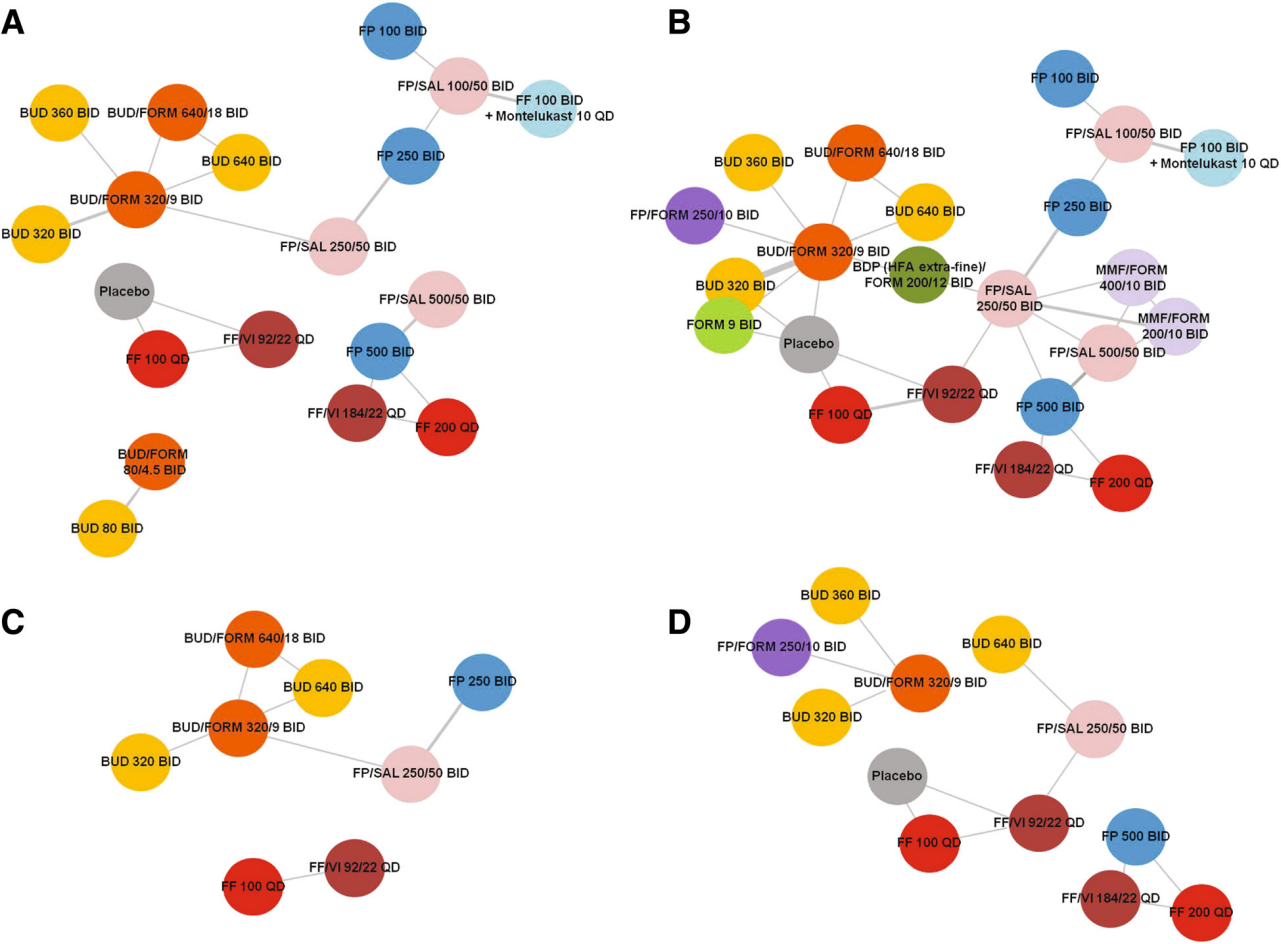
Networks of study treatments, by outcome of interest (primary analysis population). **a** change from baseline in morning PEF. **b** change from baseline in FEV_1_. **c** annual rate of moderate/severe exacerbations. **d** AQLQ Total score. *Note:* All stated doses are mcg. Delivered doses are given for FF/VI at the strengths licenced in Europe for the treatment of asthma, and for BUD/FORM. For all other treatments, nominal doses are given. Connecting lines represent studies included in the model that directly compare the two treatments. The thickness of the line is proportional to the number of studies comparing the two treatments. BDP = beclomethasone dipropionate, BID = twice daily, BUD = budesonide, F = formoterol, FEV_1_ = forced expiratory volume in 1 s, FF = fluticasone furoate, FP = fluticasone propionate, HFA = hydrofluoroalkane, MMF = mometasone furoate, QD = once daily; SAL = salmeterol, VI = vilanterol

Thirty-six unique studies were considered for inclusion in the MTC. A total of 31 parallel-group trials, with 23 different drug treatments plus placebo, were included in at least one of the four primary analyses (Fig. 1). For the primary analysis populations, eighteen studies were included in the analysis of change from baseline PEF; 28 for FEV_1_; seven for AQLQ; and six for exacerbation rates (Additional file 1: e-table 1).

For the ICS-only subset analysis, the study sets were the same for PEF and exacerbations, and slightly smaller for FEV_1_ and AQLQ (26 and six studies, respectively). The studies and treatment arms included in the MTC are summarised in Table 1. Reasons for exclusion of studies from each analysis are outlined in the Additional file 1. The networks of studies included for each outcome of interest are shown in Fig. 1 and Additional file 1: Supplementary Figure 1.

**Table 1:** Summary of studies and treatment arms included in the primary mixed treatment comparison analysis

	N (%)		N (%)
Total studies	31	Total treatment arms	75
Endpoint reported		Placebo	2 (3)
Change from baseline in FEV_1_	28 (90)	FF/VI 92/22 QD	3 (4)
Change from baseline in PEF	18 (58)	FF/VI 184/22 QD	1 (1)
Annual rate of exacerbations	6 (19)	FF 100 QD	2 (3)
Change from baseline in AQLQ	7 (23)	FF 200 QD	1 (1)
Mean age reported	41.74	FP/SAL 100/50 BID	8 (11)
Mean percent male	40.51	FP/SAL 250/50 BID	11 (15)
Mean baseline FEV_1_	2.30	FP/SAL 500/50 BID	5 (7)
		BUD/FORM 320/9 BID	12 (16)
		BUD/FORM 640/18 BID	1 (1)
		BUD/FORM 80/4.5 BID	2 (3)
		BUD 360 BID	5 (7)
		BUD 640 BID	2 (3)
		BUD 360 BID	1 (1)
		BUD 80 BID	1 (1)
		BDP (HFA extra-fine)/FORM 200/12 BID	2 (3)
		FORM 9 BID	1 (1)
		FP 250 BID	3 (4)
		FP 500 BID	4 (5)
		FP 100 BID	1 (1)
		FP 100 BID + Montelukast 10 QD	3 (4)
		FP/FORM 250/10 BID	1 (1)
		MMF/F 200/10 BID	2 (3)
		MMF/F 400/10 BID	1 (1)

*Note:* All stated doses are mcg. Delivered doses are given for FF/VI at the strengths licenced in Europe for the treatment of asthma, and for BUD/FORM. For all other treatments, nominal doses are given

*AQLQ* Asthma quality of life questionnaire, *BDP* Beclomethasone dipropionate, *BID* Twice daily, *BUD* Budesonide, *FORM* Formoterol, *FEV*_1_ Forced expiratory volume in one s, *FF* Fluticasone furoate, *FP* Fluticasone propionate, *HFA* Hydrofluoroalkane, *MMF* Mometasone furoate, *PEF* Peak expiratory flow, *QD* once daily, *SAL* Salmeterol, *TIO* Tiotropium, *VI* Vilanterol

### Primary analysis

#### Change from baseline PEF

**Fig. 2:**
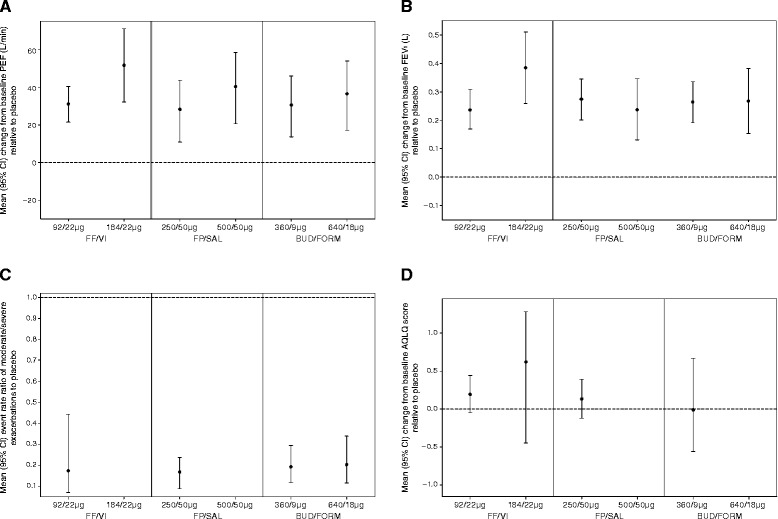
Change from baseline versus placebo for selected treatments. For studies requiring patients to be treated with ICS or ICS/LABA at baseline; full covariate analysis. **a** change from baseline in morning PEF. **b** change from baseline in FEV_1_. **c** annual rate of moderate/severe exacerbations. **d** AQLQ Total score. AQLQ = Asthma Quality of Life Questionnaire, BUD = budesonide, FORM = formoterol, FEV_1_ = forced expiratory volume in 1 s, FF = fluticasone furoate, FP = fluticasone propionate, PEF = peak expiratory flow, SAL = salmeterol, VI = vilanterol

Placebo treatment was associated with a slight mean (SD) decrease from baseline of –1.7 (8.9) l/min. All ICS/LABA combination therapies included in the model were associated with mean (SD) improvements from baseline PEF ranging from 19.65 (8.55) l/min with BUD/FORM 100/6 mcg to 49.94 (7.63) l/min with FF/VI 184/22 mcg (Fig. 2 and Additional file 1: e-table 2).

Based on a non-inferiority margin of 12 l/min, FF/VI 92/22 mcg demonstrated 97 % and 94 % probability of non-inferiority to corresponding doses of twice-daily FP/SAL 250/50 mcg and BUD/FORM 320/9 mcg, respectively (Table 2). On the same margin, FF/VI 184/22 mcg demonstrated >99 % probability of non-inferiority to corresponding doses of both FP/SAL 500/50 mcg and BUD/FORM 640/18 mcg. Based on a non-inferiority margin of 15 l/min, FF/VI 92/22 mcg demonstrated 99 % and 98 % probability of non-inferiority to corresponding doses of twice-daily FP/SAL 250/50 mcg and BUD/FORM 320/9 mcg, respectively. On the same margin, FF/VI 184/22 mcg demonstrated >99 % probability of non-inferiority to doses of both FP/SAL 500/50 mcg and BUD/FORM 640/18 mcg. Of the covariates analysed, only baseline FEV_1_ had a significant effect on PEF, though the credible interval was wide (Additional file 1: e-table 3).

**Table 2:** Posterior probability of non-inferiority for FF/VI versus other relevant ICS/LABA combination therapies*

A					
Treatment (mcg)	Comparator (mcg)	Mean difference, l (95 % CrI)	Probability of non-inferiority Margin (l/min)		
			12		15
FF/VI 92/22	FP/SAL 250/50	2.832 (−12.867–18.531)	97 %		99 %
FF/VI 92/22	BUD/FORM 320/9	0.579 (−15.155–16.312)	94 %		98 %
FF/VI 184/22	FP/SAL 500/50	11.323 (0.289–22.357)	>99 %		>99 %
FF/VI 184/22	BUD/FORM 640/18	15.136 (−0.943–31.215)	>99 %		>99 %
B					
Treatment	Comparator	Mean difference, ml (95 % CrI)	Probability of non-inferiority Margin (ml)		
			75	100	125
FF/VI 92/22	FP/SAL 250/50	−36 (−92–19)	92 %	99 %	>99 %
FF/VI 92/22	BUD/FORM 320/9	−27 (−98–45)	91 %	98 %	>99 %
FF/VI 184/22	FP/SAL 500/50	147 (48–247)	>99 %	>99 %	>99 %
FF/VI 184/22	BUD/FORM 640/18	118 (−19–255)	>99 %	>99 %	>99 %
C					
Treatment	Comparator	Rate ratio (95 % CrI)	Probability of non-inferiority Margin (event rate ratio)		
			10 %		20 %
FF/VI 92/22	FP/SAL 250/50	1.164 (0.428–3.333)	74 %		78 %
FF/VI 92/22	BUD/FORM 320/9	0.985 (0.336–2.574)	82 %		86 %
D					
Treatment	Comparator	Mean difference, units (95 % CrI)	Probability of non-inferiority Margin (units)		
			0.25		0.5
FF/VI 92/22	FP/SAL 250/50	0.060 (−0.104–0.224)	>99 %		>99 %
FF/VI 92/22	BUD/FORM 320/9	0.203 (−0.461–0.867)	90 %		96 %

*For studies requiring patients to be treated with ICS or ICS/LABA at baseline*; full covariate model

*Other relevant ICS/LABA: FP/SAL 250/50 mcg and 500/50 mcg; BUD/FORM 320/9 mcg and 640/18 mcg

**a**) change from baseline in morning PEF. **b**) change from baseline in FEV_1_. **c**) annual rate of moderate/severe exacerbations^†^. **d**) AQLQ Total score

^†^Only study length was included as a covariate in analysis of moderate/severe exacerbations data

*Note:* All stated doses are mcg

*AQLQ* Asthma quality of life questionnaire, *BUD* Budesonide, *CrI* Credible interval, *FORM* Formoterol, *FEV*_1_ Forced expiratory volume in 1 s, *FF* Fluticasone furoate, *FP* Fluticasone propionate, *PEF* Peak expiratory flow, *SAL* Salmeterol, *VI* Vilanterol

#### Change from baseline FEV_1_

All ICS/LABA combination therapies were associated with estimated improvements from baseline FEV_1_ of ≥147 ml, whereas treatment with placebo was associated with a mean (SD) decrease from baseline of 32 (58) ml. ICS/LABA treatment was associated with mean (SD) improvements from baseline, ranging from 147 (54) ml with FP/SAL 100/50 mcg to 353 (67) ml with FF/VI 184/22 mcg (Fig. 2 and Additional file 1: e-table 2).

Based on the most stringent of the three non-inferiority margins assessed, 75 ml, FF/VI 92/22 mcg demonstrated 92 % and 91 % probability of non-inferiority to corresponding doses of FP/SAL 250/50 mcg and BUD/FORM 320/9 mcg, respectively. On the same margin, FF/VI 184/22 mcg demonstrated >99 % probability of non-inferiority to corresponding doses of FP/SAL 500/50 mcg and BUD/FORM 640/18 mcg (Table 2). On a slightly less stringent margin of 100 ml, representing just under half of the MCID [13], FF/VI 92/22 mcg demonstrated 99 % and 98 % probability, respectively, of non-inferiority to corresponding doses of FP/SAL 250/50 mcg and BUD/FORM 320/9 mcg. On the same margin, FF/VI 184/22 mcg demonstrated >99 % probability of non-inferiority to corresponding doses of FP/SAL 500/50 mcg and BUD/FORM 640/18 mcg. Based on the non-inferiority margin of 125 ml, FF/VI 92/22 mcg and 184/22 mcg demonstrated >99 % probability of non-inferiority to corresponding doses of FP/SAL 250/50 mcg and 500/50 mcg and BUD/FORM 320/9 mcg and 640/18 mcg, respectively. None of the model covariates were found to have a statistically significant effect on outcomes (Additional file 1: e-table 3).

#### Annual moderate/severe exacerbation rate

Relative to a benchmark rate of 1, based upon data from the placebo arms of studies included in the model, ICS/LABA combination therapies were associated with estimated standardised event rate ratios of ≤0.203. The CrI for the reduction in event rate ratio for all active treatments excluded 1. The greatest reduction in event rate ratio relative to placebo, 0.168 (95 % CrI: 0.088–0.236), was seen with twice-daily FP/SAL 250/50 mcg. The estimated event rate ratio for once-daily FF/VI 92/22 mcg was 0.174 (95 % CrI: 0.070–0.443) (Fig. 2 and Additional file 1: e-table 2).

Because of the disconnected network for the exacerbation rate endpoint, the model included only one covariate, study duration, as the addition of further covariates resulted in the failure of the model to converge. Based on non-inferiority margins representing a 10 % and 20 % reduction in event rate, FF/VI 92/22 mcg demonstrated non-inferiority to FP/SAL 250/50 mcg with 74 % and 78 % probability, respectively. On the same margins, non-inferiority to BUD/FORM 320/9 mcg was demonstrated with 82 % and 86 % probability, respectively (Table 2). However, credible intervals for the comparisons were very wide owing to the weakness of the treatment network. No data were available for the assessment of non-inferiority of FF/VI 184/22 mcg to relevant comparators.

#### Change from baseline in AQLQ total score

All ICS/LABA combination therapies included in the model were associated with estimated mean improvements from baseline in AQLQ Total score. However, because of the limited data available for the AQLQ analysis, the CrI for all treatments were wide and did not exclude zero for the comparison of any active treatment with placebo (Fig. 3). Placebo treatment was associated with an estimated mean (SD) improvement in score of 0.233 (0.485). The greatest mean (SD) improvement from baseline for this outcome of interest, 0.854 (0.299), was observed with FF/VI 184/22 mcg (Additional file 1: e-table 2). Non-inferiority findings could not be reported for the higher strength of FF/VI; although the higher strength was in the network, none of the comparable treatments of interest were present.

Based on a non-inferiority margin of 0.25, representing half of the MCID [14], FF/VI 92/22 mcg demonstrated non-inferiority to corresponding doses of FP/SAL 250/50 mcg and BUD/FORM 320/9 mcg with >99 % and 90 % probability, respectively (Table 2). For the non-inferiority margin of 0.5, FF/VI 92/22 mcg demonstrated >99 % and 96 % probability of non-inferiority to corresponding doses of FP/SAL 250/50 mcg and BUD/FORM 320/9 mcg, respectively. None of the model covariates were found to have a statistically significant effect on outcomes (Additional file 1: e-table 3).

### ICS-only subset analysis (Table 3 and Additional file 1: e-table 4)

**Table 3:** Posterior probability of non-inferiority for FF/VI versus other relevant ICS/LABA combination therapies*

A					
Treatment	Comparator	Mean difference, ml (95 % CrI)	Probability of non-inferiority Margin (ml)		
			75	100	125
FF/VI 92/22	FP/SAL 250/50	−0.046 (−0.102–0.010)	84 %	97 %	>99 %
FF/VI 92/22	BUD/FORM 320/9	−0.037 (−0.106–0.032)	86 %	96 %	99 %
FF/VI 184/22	FP/SAL 500/50	0.163 (0.058–0.269)	>99 %	>99 %	>99 %
FF/VI 184/22	BUD/FORM 640/18	0.099 (−0.043–0.241)	99 %	>99 %	>99 %
B					
Treatment	Comparator	Mean difference, units (95 % CrI)	Probability of non-inferiority Margin (units)		
			0.25		0.5
FF/VI 92/22	FP/SAL 250/50	93 (−43–230)	>99 %		>99 %
FF/VI 92/22	BUD/FORM 320/9	89 (−502–680)	93 %		95 %

*For studies requiring patients to be treated with ICS only at baseline*

*Other relevant ICS/LABA: FP/SAL 250/50 mcg and 500/50 mcg; BUD/FORM 320/9 mcg and 640/18 mcg

**a**) change from baseline in FEV_1_. **b**) AQLQ Total score^#^

*Note:* All stated doses are mcg

^#^ For reasons of model stability, only study length was included as a covariate in analysis of moderate/severe exacerbations data

*AQLQ* Asthma quality of life questionnaire, *BUD* Budesonide, *CrI* Credible interval, *FORM* Formoterol, *FEV*_1_ Forced expiratory volume in 1 s, *FF* Fluticasone furoate, *FP* Fluticasone propionate, *SAL* Salmeterol, *VI* Vilanterol

In the ICS-only subset analysis of the FEV_1_ data, the exclusion of two studies permitting LABA use at baseline resulted in a markedly greater observed decrease from baseline FEV_1_ among patients receiving placebo. For the non-inferiority margin of 75 ml, the probabilities of non-inferiority were reduced to 84 % and 86 % for comparisons of FF/VI 92/22 mcg with FP/SAL 250/50 mcg and BUD/FORM 400/12 mcg, respectively. The other probabilities of non-inferiority all remained higher than 96 %. In the PEF and exacerbations analyses, no studies were excluded in the ICS-only subset analysis for annual moderate/severe exacerbation rate, so the outcomes were unchanged from those of the main analysis.

The ICS-only subset analysis of the AQLQ data excluded one of the seven studies that were included in the main analysis. As a consequence of non-convergence owing to the weakness of the network of six studies, the full covariate model could not be fit. A model including only the study duration covariate was fit. The resulting probability of non-inferiority of FF/VI 92/22 mcg to BUD/FORM 320/9 mcg, on a non-inferiority margin of 0.25, was 93 % and the probability of non-inferiority to FP/SAL 250/50 mcg of >99 % was unchanged. The mean (SD) improvement observed with placebo, 0.177 (0.414), was lower than in the main analysis. The CrI for the treatment difference vs placebo on the AQLQ outcome excluded zero for both strengths of FF/VI and also for high-dose FP and FF monotherapy; for the remaining treatments in the network, all other comparisons vs placebo included zero.

### Sensitivity analysis (Additional file 1: e-table 5)

Sensitivity analyses, in which additional studies that did not fulfil the primary analysis inclusion criteria were added to the network, were conducted for the PEF and exacerbations outcomes. For PEF, the addition of nine studies which did not report change from baseline over the study duration up to study end to the 18 included in the primary analysis resulted in reductions in the reported probabilities of non-inferiority of FF/VI 92/22 mcg vs FP/SAL 250/50 mcg and BUD/FORM 320/9 mcg, whereas the results for comparisons involving 184/22 mcg were similar to those of the primary analysis. For exacerbations, the addition of six studies that withdrew subjects and discontinued follow-up after an exacerbation to the six included in the primary analysis resulted in a narrowing of the credible intervals for comparisons of FF/VI 92/22 mcg and increased probabilities of non-inferiority vs FP/SAL 250/50 mcg and BUD/FORM 320/9 mcg. It was also possible, using the sensitivity analysis network, to report a probability of non-inferiority for FF/VI 184/22 mcg vs BUD/FORM 640/18 mcg on exacerbations; however, the CrI for this estimate is wide. Thus, the sensitivity analysis demonstrates that the results for the exacerbations network are susceptible to the studies that are included in the network. The findings of a separate sensitivity analysis in which the exacerbations analysis was rerun with alternate person-years definitions were similar to those of the primary analysis.

### Assessment of alternative modelling approaches (Additional file 1: e-table 6)

The findings and details of the post-hoc analysis utilising alternative modelling approaches–specifically, a frequentist analysis using a random effects model with fixed study and treatment effects, and a pairwise contrast analysis (FEV_1_ endpoint only)–are reported in Additional file 1: e-table 6. The results of these analyses showed that, where the application of the varied methodologies to the dataset was feasible, PEF, FEV_1_ and AQLQ results using these methodologies were consistent with those of the primary MTC analyses.

## Discussion

FF/VI represents the first once-daily ICS/LABA combination to be approved for use in the treatment of asthma; two strengths of FF/VI have been approved in Europe and Japan. We sought to compare the clinical efficacy of FF/VI 92/22 mcg with that of twice-daily FP/SAL 250/50 mcg and BUD/FORM 320/9 mcg, and that of FF/VI 184/22 mcg with FP/SAL 500/50 mcg and BUD/FORM 640/18 mcg. FF/VI 92/22 mcg has previously been shown to be comparable in efficacy in improving lung function and health status with FP/SAL 250/50 mcg in a head-to-head randomised controlled trial [7].

Using an MTC approach, we examined the probability of non-inferiority of once-daily FF/VI compared with corresponding strengths of twice-daily FP/SAL and BUD/FORM by combining data on clinical efficacy outcomes from several RCTs. All three ICS/LABA combination therapies have previously been shown to be associated with improvements in these outcomes relative to placebo.

We chose to use a Bayesian, hierarchical MTC modelling approach to synthesise evidence from RCTs conducted in adolescents and adults with asthma that involved at least one ICS/LABA comparator. Broad-scope searches were used to identify as many studies as possible that were potentially suitable for inclusion in the MTC. Exclusion criteria were subsequently applied on an outcome-by-outcome basis. The Bayesian approach that we used enabled a probabilistic estimate of non-inferiority to be generated directly from the posterior distribution [22].

To output meaningful probabilities of non-inferiority, it was necessary to select suitable margins. The non-inferiority margins used for the PEF analysis in this study, 12 l/min and 15 l/min, are well-established margins used in numerous previous studies and based upon European Medicines Agency guidelines [11, 23, 24]. For FEV_1_, the margins of 75 ml, 100 ml and 125 ml were chosen based upon the minimal clinically important difference (MCID) of 230 ml [13] and non-inferiority margins used in previous comparative studies involving FF/VI [6] or FF [25]. For exacerbations, the non-inferiority margins of 10 % and 20 % rate ratio reductions are consistent with the margin of 1.18 used in a previous non-inferiority study of FP/SAL vs FP [12]. The accepted MCID for the AQLQ is 0.5 [14], hence the use of a margin of 0.25, representing half of the MCID, preserves 50 % of the active comparator effect. We consider that, as these margins are smaller than the MCID and therefore imply higher thresholds for demonstrating non-inferiority between treatments, they represent conservative margins for the non-inferiority analysis. However, it is important to note that a finding of a low probability of non-inferiority, using such conservative margins, does not imply lack of comparability or inferiority.

Based on these conservative margins, the results of the primary MTC analyses suggest that there is a high probability that FF/VI 92/22 mcg is non-inferior to FP/SAL 250/50 mcg and BUD/FORM 320/9 mcg on lung function (PEF and FEV_1_) and health status (AQLQ) endpoints, supporting the findings of the previous head-to-head RCT of FF/VI 92/22 mcg compared with FP/SAL 250/50 mcg. The analysis of exacerbation rate was inconclusive owing to the lack of sufficient data and disconnectedness of the network.

The effectiveness of any meta-analytic method can be limited by the amount of clinical trial data available for each of the treatments in the analysis. As a consequence of the limited number of RCTs available to inform comparisons of ICS/LABA therapies in asthma, the CrI for most of the non-inferiority estimates in our study were wide. Moreover, we decided *a priori* to treat different strengths of the same therapies as different treatments. This represents both a strength–in that the comparisons we have studied are more precise–and a weakness of our approach, as combining dosages may have produced a stronger and more connected network for the lung function and health status outcomes. The CrI were particularly wide for the outcomes of secondary interest (i.e. exacerbations and AQLQ). Exacerbations, in particular, are relatively rare events and are therefore typically only examined in longer-term studies. In addition, the primary endpoints of most RCTs in asthma assess lung function, rather than exacerbations. As such, our study network was weak with respect to exacerbations; this MTC incorporated only six studies, including just one study of FF/VI 92/22 mcg [5] and no studies of FF/VI 184/22 mcg for the primary exacerbations analysis. The small number of studies led to limited information in the network and subsequent disconnections in treatment networks. The full model did not converge because of the weak network. One consequence of this was that we were unable to account for covariates in the exacerbations MTC as was possible for the other three MTC outcomes. The *post-hoc* assessment of alternative modelling approaches showed that the findings of the lung function and health status MTCs were consistent upon the application of varied methodologies, including a frequentist approach, to the data where the network was sufficiently connected to permit this.

Population and endpoint heterogeneity in the studies included in the MTC analyses was addressed as far as possible by our inclusion criteria to ensure that patient populations were suitable for comparison. To minimise study-to-study variability, studies were only included in the primary PEF analysis if they reported mean change from baseline averaged across the whole trial, rather than from baseline to a specific timepoint. For the same reason, studies were excluded from the exacerbations analysis if the definition of exacerbation differed considerably from that set out in the ATS/ERS Task Force recommendation [8]. The findings of sensitivity analyses of both of these outcomes, conducted using data from enlarged networks derived from relaxed inclusion criteria, suggest that the MTC findings are highly susceptible to the addition of studies to the network.

Despite the measures we took in order to reduce study-to-study variability as far as possible given the nature of the analysis, over-dispersion was observed in the primary analysis model distributions. Through study-level covariate modelling within the MTC, we assessed whether variables including study duration, average patient age and exacerbation history affected the comparisons; we sought to correct for this through the incorporation of heterogeneity factors at the study level. Significant covariate effects of baseline FEV_1_ and study duration were observed in the PEF analysis only. The observation that greater baseline FEV_1_ is associated with slightly greater improvement from baseline in PEF is consistent with incompleteness of reversibility in patients with more severe airflow limitation [26].

Still, a limitation of this approach that should be borne in mind in the interpretation of the results is that it is not possible to correct for all confounding factors, particularly as patient-level data were not available. For instance, although the study inclusion criteria specified that patients had to be uncontrolled/symptomatic at baseline, it was not possible to ensure a consistent definition of “uncontrolled” across studies as the required data was often not reported. Furthermore, the fact that we have synthesised evidence from trials conducted at different periods of time and in different regions means that there will inevitably be some degree of underlying variation that could have influenced the findings. For instance, basic standards of care vary across regions and typically improve over time.

Although MTC are established as a useful tool for the synthesis of evidence that is suitable for use in clinical decision-making [27], to our knowledge, this is the first study to apply this methodology to data from clinical trials in asthma to investigate the relative efficacy of specific treatments. In a recently-published network meta-analysis in which data on treatment interventions in asthma were combined across classes [28] and their effectiveness assessed, ICS/LABA combination therapies were found to be the most effective intervention for the prevention of asthma exacerbations. In COPD, findings of meta-analyses of treatment efficacy data [29, 30] have indicated that ICS/LABA combination therapy have a greater positive effect on COPD outcomes than alternative treatment modalities.

## Conclusions

The findings of the MTC suggest that the efficacy of both strengths of once-daily FF/VI in asthma is broadly comparable to that of corresponding doses of established twice-daily ICS/LABA combinations, FP/SAL and BUD/FORM, on lung function and health status outcomes of interest in the primary study populations. The MTC supported the findings of a previously-reported head-to-head randomised controlled trial of FF/VI 92/22 mcg vs FP/SAL 250/50 mcg in which it was shown that the efficacy of these treatments in improving lung function and health status endpoints is similar [7]. It should be borne in mind that the MTC findings are obtained through the analysis of outcomes from RCTs and any potential efficacy benefits that may derive from treatment attributes such as once- vs twice-daily dosing in real-world clinical practice will not be reflected in these data.

### Supplementary data update

The mixed treatment comparison was updated with more recently published data; studies published between 18th December 2012 and 30th July 2014 were identified by an update of the systematic review. Seven additional studies were identified for inclusion in this update, thus a total of 38 studies were included and are presented in the study supplement (Additional file 2: Tables S1-S3). The results including these data are in line with those of the original analyses, and thus did not affect any of the conclusions previously drawn.

## Additional files

Additional file 1:
**e-table 1:** Characteristics of the studies and treatment arms included in the MTC. **e-table 2:** Results of mixed treatment comparisons for ICS/LABA treatments of interest (full covariate model, if available). **e-table 3:** Summary of findings of covariate analysis, by outcome of interest (primary analysis population; full covariate model, if available). **e-table 4:** Results of mixed treatment comparisons by ICS/LABA treatment. **e-table 5:** Posterior probability of non-inferiority for FF/VI versus other relevant ICS/LABA combination therapies*, sensitivity analysis. **e-table 6:** Outcomes of assessment of alternative modelling approaches. **Supplementary figure 1:** Networks of study treatments, by outcome of interest (sensitivity analysis population). (PDF 705 kb)
Additional file 2:
**Table S1:** Summary of studies and treatment arms included in the updated primary mixed treatment comparison analysis. **Table S2:** Posterior probability of non-inferiority for FF/VI versus other relevant ICS/LABA combination therapies **Table S3:** Posterior probability of non-inferiority for FF/VI versus other relevant ICS/LABA combination therapies. (DOCX 22 kb)

## Abbreviations

AQLQ: Asthma quality of life questionnaire
ATS: American thoracic society
BUD: Budesonide
CrI: Credible interval
ERS: European respiratory society
FORM: Formoterol
FEV_1_: Forced expiratory volume in 1 s
FF: Fluticasone furoate
FP: Fluticasone propionate
ICS: Inhaled corticosteroid
LABA: Long-acting beta_2_ agonist
MCID: Minimum clinically important difference
MTC: Mixed treatment comparison
PEF: Peak expiratory flow
RCT: Randomised controlled trial
SAL: Salmeterol
SD: Standard deviation
VI: Vilanterol
