# Supplementary material for: Once-daily fluticasone furoate/vilanterol versus twice daily combination therapies in asthma–mixed treatment comparisons of clinical efficacy
Source: Asthma Res Pract. 2016 Feb 8;2:4. doi: 10.1186/s40733-015-0016-0 (PMC5142397; doi:10.1186/s40733-015-0016-0)
Supplement: Additional file 1: — e-table 1: Characteristics of the studies and treatment arms included in the MTC. e-table 2: Results of mixed treatment comparisons for ICS/LABA treatments of interest (full covariate model, if available). e-table 3: Summary of findings of covariate analysis, by outcome of interest (primary analysis population; full covariate model, if available). e-table 4: Results of mixed treatment comparisons by ICS/LABA treatment. e-table 5: Posterior probability of non-inferiority for FF/VI versus other relevant ICS/LABA combination therapies*, sensitivity analysis. e-table 6: Outcomes of assessment of alternative modelling approaches. Supplementary figure 1: Networks of study treatments, by outcome of interest (sensitivity analysis population). (PDF 705 kb) [file 40733_2015_16_MOESM1_ESM.pdf]

e-Appendix

e-Table 1 Characteristics of the studies and treatment arms included in the MTC

Study	Regimen	Endpoint, weeks measured				Mean Age, years	Male, %	Baseline Mean FEV_1, L
		FEV_1	PEF	Exac.	AQLQ			
Aubier 1999	FP 500 BID	28	12			50.00	52.73	2.33
	FP/SAL 500/50 BID	28	12			48.00	50.29	2.44
	FP/SAL 500/50 BID	28	12			46.00	57.49	2.32
Bateman 1998	FP/SAL 100/50 BID	12	12			33.00	41.00	2.33
	FP/SAL 100/50 BID	12	12			33.00	44.00	2.42
Bateman 2001	FP/SAL 100/50 BID	12	12			38.60	47.00	2.37
	FP 100 BID	12	12			39.50	41.00	2.44
	FP/SAL 100/50 BID	12	12			40.70	44.00	2.47
Bergmann 2004	FP/SAL 250/50 BID	12	12			49.80	49.40	2.33
	FP 500 BID	12	12			48.90	43.50	2.35
Bernstein 2011	FP/SAL 250/50 BID	12	12			45.10	37.00	2.37
	MMF/FORM 200/10 BID	12	12			44.80	36.00	2.30
Bodzenta-Lukaszyk 2012	BUD/FORM 320/9 BID	12	12	12	12	48.10	27.30	1.90
	FP/FORM 250/10 BID	12	12	12	12	49.80	37.10	1.94
Brown 2012	BUD/FORM 320/9 BID	52	52	52		36.20	33.95	2.31
	BUD 320 BID	52	52	52		38.40	36.44	2.31
Chapman 1999	FP/SAL 250/50 BID	12	12			41.40	43.00	2.55

	FP/SAL 250/50 BID	12	12			42.80	51.00	2.51
Condemi 1999	FP 250 BID	24	24			36.80	40.00	2.14
	FP/SAL 100/50 BID	24	24			36.90	38.00	2.12
Dahl 2006	BUD/FORM 320/9 BID	24	24	24		45.60	44.00	2.40
	FP/SAL 250/50 BID	24	24	24		47.10	41.00	2.43
Busse 2013 (GlaxoSmithKline HZA106839)*	FF/VI 100/25 QD			52		39.70	35.00	2.31
	FF/VI 200/25 QD			52		38.50	39.00	2.29
	FP 500 BID			52		38.60	38.00	2.35
Bleecker 2014 (GlaxoSmithKline HZA106827)	Placebo	12	12	12	12	38.10	45.00	2.33
	FF 100 QD	12	12	12	12	40.40	39.00	2.29
	FF/VI 92/22 QD	12	12	12	12	40.70	42.00	2.34
O'Byrne 2013 (GlaxoSmithKline HZA106829)	FF 200 QD	24	24	24	24	44.60	42.00	2.19
	FF/VI 184/22 QD	24	24	24	24	44.60	41.00	2.13
	FP 500 BID	24	24	24	24	47.30	41.00	2.14
Bateman 2013 (GlaxoSmithKline HZA106837)	FF/VI 92/22 QD	76		50		42.30	32.00	2.22
	FF 100 QD	76		50		41.10	34.00	2.19
Woodcock 2013 (GlaxoSmithKline HZA113091)	FF/VI 92/22 QD	24		24	24	43.80	39.00	2.01
	FP/SAL 250/50 BID	24		24	24	41.90	39.00	2.05
Jenkins 2000	BUD 640 BID				12			

	FP/SAL 250/50 BID				12			
Katial 2011	FP 250 BID	52	52	52		39.30	36.00	2.30
	FP/SAL 250/50 BID	52	52	52		36.80	38.00	2.36
Kerwin 2011	FP 250 BID	52	52	52		39.60	43.00	2.53
	FP/SAL 250/50 BID	52	52	52		40.90	40.00	2.53
Kuna 2006	BUD/FORM 80/4.5 BID		12			45.10	43.96	2.32
	BUD/FORM 80/4.5 BID		12			45.80	40.10	2.36
	BUD 80 BID		12			43.90	37.68	2.36
Maspero 2010	MMF/FORM 200/10 BID	52				32.70	34.75	2.65
	FP/SAL 250/50 BID	52				32.40	44.12	2.62
	MMF/FORM 400/10 BID	52				39.30	33.85	2.31
	FP/SAL 500/50 BID	52				37.10	38.46	2.41
Morice 2007	BUD/FORM 320/9 BID	12			12	39.00	38.86	2.09
	BUD 320 BID	12			12	40.00	40.17	2.01
	BUD/FORM 320/9 BID	12			12	40.00	31.34	2.07
Nelson 2000	FP 100 BID + Montelukast 10 QD	12	12			40.20	39.00	2.38
								2.39
	FP/SAL 100/50 BID	12	12			43.00	40.00	
Noonan 2006	BUD 320 BID	12	12			41.80	35.50	2.30

	BUD/FORM 320/9 BID	12	12			40.30	43.50	2.23
	BUD/FORM 320/9 BID	12	12			40.70	34.90	2.23
	FORM 9 BID	12	12			40.00	35.00	2.19
	Placebo	12	12			41.90	42.40	2.29
OPTIMA trial*	BUD 80 BID		52					
	BUD/FORM 80/4.5 BID		52					
	BUD 160 BID		52					
	BUD/FORM 160/4.5 BID		52					
Papi 2007	BDP (HFA extra-fine) /FORM 200/12 BID	12	12			47.30	45.20	2.08
	FP/SAL 250/50 BID	12	12			49.70	42.50	1.98
Papi 2007	BDP (HFA extra-fine) /FORM 200/12 BID	12	12			43.40	42.10	2.30
	BUD/FORM 320/9 BID	12	12			46.00	42.20	2.21
Pavord 2007	FP 100 BID + Montelukast 10 QD	12	12			34.40	58.00	2.76
	FP/SAL 100/50 BID	12	12			36.30	45.00	2.65
Peters 2008	BUD/FORM 640/18 BID	52	52	52		41.00	37.00	2.44
	BUD/FORM 320/9 BID	52	52	52		38.60	40.90	2.44
	BUD 640 BID	52	52	52		39.80	31.60	2.34
Ringdal 2003	FP 100 BID + Montelukast 10 QD		12	12		43.00	45.00	2.41
	FP/SAL 100/50 BID		12	12		43.00	46.00	2.47

Spector 2012	BUD 320 BID	12	12		12	38.60	28.80	2.04
	BUD/FORM 320/9 BID	12	12		12	39.80	41.20	2.01
Stirbulov 2012	BUD 320 BID	12	12					2.25
	BUD/FORM 320/9 BID	12	12					2.32
Van 2001	FP 500 BID	12	12			46.00	42.00	2.23
	FP/SAL 500/50 BID	12	12			48.00	40.00	2.09
	FP/SAL 500/50 BID	12	12			47.00	40.00	2.24
Zangrilli 2011	BUD/FORM 320/9 BID	12	12			37.00	35.00	2.20
	BUD 320 BID	12	12			39.80	33.90	2.22

Note: All stated doses are µg. Delivered doses are given for FF/VI at the strengths licenced in Europe for the treatment of asthma, and for BUD/FORM. For all other treatments, nominal doses are given.

* Study excluded from primary analysis

AQLQ = Asthma Quality of Life Questionnaire. BDP = beclomethasone dipropionate, BID = twice daily, BUD = budesonide, FORM = formoterol, FEV_1 = forced expiratory volume in one second, FF = fluticasone furoate, FP = fluticasone propionate, MMF = mometasone furoate, PEF = peak expiratory flow, QD = once daily; SAL = salmeterol, TIO = tiotropium, VI = vilanterol

e-Table 2 Results of mixed treatment comparisons for ICS/LABA treatments of interest (full covariate model, if available)

For studies requiring patients to be treated with ICS or ICS/LABA at baseline

A: change from baseline in morning PEF; B: change from baseline in FEV_1; C: annual rate of moderate/severe exacerbations[†]; D: AQLQ Total score

A

Treatment (mcg)	Change from baseline PEF, L/min	
	Mean change from baseline (SD)	Mean difference from placebo (95% CrI)
Placebo	–1.745 (8.925)	–
FF/VI 184/22	49.942 (7.631)	51.688 (32.174, 71.166)
FF/VI 92/22	29.375 (8.722)	31.120 (21.530, 40.506)
FP/SAL 500/50	38.619 (6.929)	40.365 (20.606, 58.488)
FP/SAL 250/50	26.542 (3.655)	28.288 (10.818, 43.635)
FP/SAL 100/50	32.069 (5.201)	33.814 (17.854, 49.208)
BUD/FORM 640/18	34.806 (4.795)	36.552 (16.990, 53.975)
BUD/FORM 320/9	28.796 (3.735)	30.541 (13.626, 46.024)
BUD/FORM 160/9	–	–
BUD/FORM 80/4.5	19.650 (8.545)	21.395 (–0.105, 42.498)
BDP/FORM 200/12	–	–
FP/FORM 250/10	–	–
MMF/FORM 400/10	–	–
MMF/FORM 200/10	–	–

B

Treatment (mcg)	Change from baseline FEV_1, mL	
	**Mean change from baseline (SD)**	**Mean difference from placebo (95% CrI)**
Placebo	–32 (58)	-
FF/VI 184/22	353 (67)	385 (259, 510)
FF/VI 92/22	205 (47)	237 (169, 309)
FP/SAL 500/50	206 (51)	238 (131, 346)
FP/SAL 250/50	241 (42)	274 (201, 345)
FP/SAL 100/50	147 (54)	179 (75, 279)
BUD/FORM 640/18	235 (56)	267 (154, 382)
BUD/FORM 320/9	232 (51)	264 (192, 335)
BUD/FORM 160/9	–	–
BUD/FORM 80/4.5	–	–
BDP/FORM 200/12	258 (62)	291 (185, 394)
FP/FORM 250/10	205 (72)	237 (112, 362)
MMF/FORM 400/10	167 (66)	199 (50, 357)
MMF/FORM 200/10	192 (50)	224 (132, 315)

C

Treatment (mcg)	Annual exacerbation rate<br><br>Mean event rate ratio to placebo<br>(95% CrI)
Placebo	1
FF/VI 184/22	–
FF/VI 92/22	0.174 (0.070, 0.443)
FP/SAL 500/50	–
FP/SAL 250/50	0.168 (0.088, 0.236)
FP/SAL 100/50	–
BUD/FORM 640/18	0.203 (0.115, 0.339)
BUD/FORM 320/9	0.193 (0.116, 0.294)
BUD/FORM 160/9	–
BUD/FORM 80/4.5	–
BDP/FORM 200/12	–
FP/FORM 250/10	–
MMF/FORM 400/10	–
MMF/FORM 200/10	–

[†]For reasons of model stability, only study length was included as a covariate in analysis of moderate/severe exacerbations data.

D

Treatment (mcg)	Change from baseline AQLQ Total score, units	
	**Mean change from baseline (SD)**	**Mean difference from placebo (95% CrI)**
Placebo	0.233 (0.485)	–
FF/VI 184/22	0.854 (0.299)	0.621 (–0.445, 1.280)
FF/VI 92/22	0.427 (0.424)	0.194 (–0.048, 0.443)
FP/SAL 500/50	–	–
FP/SAL 250/50	0.367 (0.443)	0.134 (–0.118, 0.390)
FP/SAL 100/50	–	–
BUD/FORM 640/18	–	–
BUD/FORM 320/9	0.224 (0.708)	–0.009 (–0.557, 0.667)
BUD/FORM 160/9	–	–
BUD/FORM 80/4.5	–	–
BDP/FORM 200/12	–	–
FP/FORM 250/10	0.175 (0.600)	–0.058 (–0.569, 0.440)
MMF/FORM 400/10	–	–
MMF/FORM 200/10	–	–

Note: All stated doses are µg.

AQLQ = Asthma Quality of Life Questionnaire, BDP = beclomethasone dipropionate, BUD = budesonide, CrI = credible interval, FORM = formoterol, FEV_1 = forced expiratory volume in one second, FF = fluticasone furoate, FP = fluticasone propionate, MMF = mometasone

furoate, PEF = peak expiratory flow, SAL = salmeterol, SD = standard deviation, VI = vilanterol

e-Table 3 Summary of findings of covariate analysis, by outcome of interest (primary analysis population; full covariate model, if available)

For studies requiring patients to be treated with ICS or ICS/LABA at baseline; full covariate analysis

A: change from baseline in morning PEF; B: change from baseline in FEV_1; C: annual rate of moderate/severe exacerbations[†]; D: AQLQ Total score

A

Covariate	Mean (SD)	95% CrI
Age (age – 40)	0.192 (0.493)	-0.774, 1.157
Male (proportion males – 0.4)	-0.454 (0.270)	-0.984, 0.076
FEV_1 at baseline (L) (FEV_1 – 2.4)	26.259 (11.924)*	2.887, 49.630
Study length		
≤20 weeks	5.347 (5.111)	-4.669, 15.364
>20–≤40 weeks	13.621 (6.233)	1.404, 25.838
>40–≤60 weeks	Reference	Reference
>60 weeks	-	-

* Significant

B

**Covariate**	**Mean (SD)**	**95% CrI**
Age (age – 40)	0.001 (0.005)	-0.010, 0.011
Male (proportion males – 0.4)	-0.002 (0.002)	-0.005, 0.002
FEV_1 at baseline (L) (FEV_1 – 2.4)	0.059 (0.093)	-0.123, 0.241
Study length		
≤20 weeks	0.041 (0.055)	-0.066, 0.149
>20–≤40 weeks	0.079 (0.061)	-0.041, 0.199
>40–≤60 weeks	Reference	Reference
>60 weeks	0.073 (0.114)	-0.151, 0.296

C

**Covariate**	**Mean (SD)**	**Rate ratio (95% CrI)**
Study length		
≤20 weeks	–	–
>20–≤40 weeks	0.109 (0.490)	1.311 (0.427, 2.911)
>40–≤60 weeks	Reference	Reference
>60 weeks	–	–

†For reasons of model stability, only study length was included as a covariate in analysis of moderate/severe exacerbations data.

D

**Covariate**	**Mean (SD)**	**95% CrI**
Age (age – 40)	0.012 (0.033)	-0.052, 0.077
Male (proportion males – 0.4)	0.005 (0.013)	-0.021, 0.030
FEV_1 at baseline (L) (FEV_1 – 2.4)	-0.051 (1.174)	-2.352, 2.250
Study length		
≤20 weeks	0.436 (0.332)	-0.214, 1.085
>20–≤40 weeks	–	–
>40–≤60 weeks	Reference	Reference
>60 weeks	–	–

AQLQ = Asthma Quality of Life Questionnaire, BID = twice daily, CrI = credible interval, FEV_1 = forced expiratory volume in one second. PEF = peak expiratory flow, SD = standard deviation

e-Table 4 Results of mixed treatment comparisons by ICS/LABA treatment

For studies requiring patients to be treated with ICS only at baseline, full covariate analysis

A: change from baseline in FEV_1; B: AQLQ Total score

A

Treatment (mcg)	Change from baseline FEV_1, mL	
	Mean change from baseline (SD)	Mean difference from placebo (95% CrI)
Placebo	–106 (51)	-
FF/VI 184/22	267 (65)	374 (245, 504)
FF/VI 92/22	123 (47)	230 (159, 297)
FP/SAL 500/50	104 (55)	211 (99, 323)
FP/SAL 250/50	169 (37)	275 (203, 347)
FP/SAL 100/50	87 (46)	194 (87, 297)
BUD/FORM 640/18	168 (52)	274 (163, 385)
BUD/FORM 320/9	160 (40)	267 (194, 339)
BUD/FORM 160/9	-	-
BUD/FORM 80/4.5	-	-
BDP/FORM 200/12	190 (53)	297 (189, 403)
FP/FORM 250/10	120 (70)	227 (101, 353)

B

Treatment (mcg)	Change from baseline AQLQ Total score, units	
	**Mean change from baseline (SD)**	**Mean difference from placebo (95% CrI)**
Placebo	0.177 (0.414)	-
FF/VI 184/22	0.850 (0.393)	0.673 (0.054, 1.621)
FF/VI 92/22	0.381 (0.403)	0.204 (0.028, 0.382)
FP/SAL 500/50	-	-
FP/SAL 250/50	0.288 (0.402)	0.111 (–0.109, 0.337)
FP/SAL 100/50	-	-
BUD/FORM 640/18	-	-
BUD/FORM 320/9	0.293 (0.267)	0.115 (–0.306, 1.155)
BUD/FORM 160/9	-	-
BUD/FORM 80/4.5	-	-
BDP/FORM 200/12	-	-
FP/FORM 250/10	0.333 (0.286)	0.156 (–0.317, 1.163)

Note: All stated doses are µg.

AQLQ = Asthma Quality of Life Questionnaire, BDP = beclamethasone dipropionate, BUD = budesonide, CrI = credible interval, FORM = formoterol, FEV_1 = forced expiratory volume in one second, FF = fluticasone furoate, FP = fluticasone propionate, SAL = salmeterol, VI = vilanterol

e-Table 5 Posterior probability of non-inferiority for FF/VI versus other relevant ICS/LABA combination therapies*, sensitivity analysis

For studies requiring patients to be treated with ICS or ICS/LABA at baseline; full covariate analysis

*Other relevant ICS/LABA: FP/SAL 250/50mcg and 500/50mcg; BUD/FORM 320/9mcg and 640/18mcg

A: change from baseline in PEF; B: annual rate of moderate/severe exacerbations[#]

A

**Treatment (mcg)**	**Comparator (mcg)**	**Mean difference, L (95% CrI)**	**Probability of non-inferiority Margin (L/min)**	
			**12**	**15**
FF/VI 92/22	FP/SAL 250/50	–10.57 (–23.14, 2.01)	59%	76%
FF/VI 92/22	BUD/FORM 320/9	–11.42 (–23.98, 1.14)	54%	71%
FF/VI 184/22	FP/SAL 500/50	13.85 (2.68, 25.03)	>99%	>99%
FF/VI 184/22	BUD/FORM 640/18	12.71 (–2.05, 27.46)	>99%	>99%

B

**Treatment**	**Comparator**	**Rate ratio (95% CrI)**	**Probability of non-inferiority Margin (event rate ratio)**	
			**10%**	**20%**
FF/VI 92/22	FP/SAL 250/50	0.62 (0.29, 1.18)	96%	98%
FF/VI 92/22	BUD/FORM 320/9	0.47 (0.20, 0.90)	99%	100%
FF/VI 184/22	BUD/FORM 640/18	0.43 (0.09, 1.37)	95%	96%

Note: All stated doses are μg.

[#] For reasons of model stability, only study length was included as a covariate in analysis of moderate/severe exacerbations data.

AQLQ = Asthma Quality of Life Questionnaire, BUD = budesonide, CrI = credible interval, FORM = formoterol, FEV_1 = forced expiratory volume in one second, FF = fluticasone furoate, FP: fluticasone propionate, SAL = salmeterol; VI = vilanterol

eTable 6 Outcomes of assessment of alternative modelling approaches ([1] full covariate random study effects model, [2] No covariates random effects contrast based model, [3] full covariate fixed study effects model)

A: change from baseline in morning PEF*; B: change from baseline in FEV_1; C: annual rate of moderate/severe exacerbations*; D: AQLQ Total score*

*Note: Contrast-based analysis was not possible for this outcome because the network was insufficiently connected

A

Treatment (mcg)	Comparator (mcg)	Mean difference (95% CrI) [1]	Probability of non-inferiority		Fixed study effects [3]	p-value [3]
			Pr (A~B|12 l/min)	Pr (A~B|15 l/min)		
FF/VI 92/22	FP/SAL 250/50	2.832 (-12.867, 18.531)	97%	99%	1.613 (-15.712, 18.938)	0.855
FF/VI 92/22	BUD/FORM 320/9	0.579 (-15.155, 16.312)	94%	98%	0.161 (-16.751, 17.072)	0.985
FF/VI 184/22	FP/SAL 500/50	11.323 (0.289, 22.357)	>99%	>99%	18.112 (-2.969, 39.192)	0.092
FF/VI 184/22	BUD 640/18	15.136 (-0.943, 31.215)	>99%	>99%	11.800 (-9.152, 32.752)	0.270

B

<table>
<tr><th rowspan="2">Treatment</th><th rowspan="2">Comparator</th><th rowspan="2">Mean difference (95% CrI) [1]</th><th colspan="3">Probability of non-inferiority</th><th rowspan="2">geMTC [2]</th><th rowspan="2">Fixed study effects [3]</th><th rowspan="2">p-value [3]</th></tr>
<tr><th>Pr (A~B| 75mL)</th><th>Pr (A~B| 100mL)</th><th>Pr (A~B| 125mL)</th></tr>
<tr><td>FF/VI 92/22</td><td>FP/SAL 250/50</td><td>-0.036 (-0.092, 0.019)</td><td>92%</td><td>99%</td><td>>99%</td><td>-0.09 (-0.23, 0.04)</td><td>0.016 (-0.187, 0.219)</td><td>0.877</td></tr>
<tr><td>FF/VI 92/22</td><td>BUD/FORM 320/9</td><td>-0.027 (-0.098, 0.045)</td><td>91%</td><td>98%</td><td>>99%</td><td>-0.10 (-0.26, 0.05)</td><td>-0.021 (-0.265, 0.224)</td><td>0.869</td></tr>
<tr><td>FF/VI 184/22</td><td>FP/SAL 500/50</td><td>0.147 (0.048, 0.247)</td><td>>99%</td><td>>99%</td><td>>99%</td><td>0.15 (-0.07, 0.36)</td><td>0.264 (0.023, 0.504)</td><td>0.032</td></tr>
<tr><td>FF/VI 184/22</td><td>BUD/FORM 640/18</td><td>0.118 (-0.019, 0.255)</td><td>>99%</td><td>>99%</td><td>>99%</td><td>0.20 (-0.12, 0.51)</td><td>0.027 (-0.632, 0.685)</td><td>0.938</td></tr>
</table>

C

**Treatment (mcg)**	**Comparator (mcg)**	**Event rate ratio A/B (95% CrI)**	**Probability of non-inferiority**		**Fixed study effects [3]**	**p-value [3]**
			**Pr (A~B|0.10)**	**Pr (A~B|0.20)**		
FF/VI 92/22	FP/SAL 250/50	1.164 (0.428, 3.333)	74%	78%	0.737 (0.578, 0.939)	0.014
FF/VI 92/22	BUD/FORM 320/9	0.985 (0.336, 2.574)	82%	86%	0.805 (0.616, 1.053)	0.114
FF/VI 184/22	FP/SAL 500/50	NA	NA	NA	NA	
FF/VI 184/22	BUD/FORM 640/18	NA	NA	NA	NA	

† For reasons of model stability, only study length was included as a covariate in analysis of moderate/severe exacerbations data.

D

**Treatment (mcg)**	**Comparator (mcg)**	**Mean difference (95% CrI) [1]**	**Probability of non-inferiority**		**Fixed study effects [3]**	**p-value [3]**
			**Pr (A~B|0.25)**	**Pr (A~B|0.5)**		
FF/VI 92/22	FP/SAL 250/50	0.060 (-0.104, 0.224)	>99%	>99%	0.038 (-0.595, 0.670)	0.907
FF/VI 92/22	BUD/FORM 320/9	0.203 (-0.461, 0.867)	90%	96%	NA	
FF/VI 184/22	FP/SAL 500/50	NA	NA	NA	NA	
FF/VI 184/22	BUD/FORM 640/18	NA	NA	NA	NA	

Note: All stated doses are μg.

AQLQ = Asthma Quality of Life Questionnaire, BDP = beclamethasone dipropionate, BUD = budesonide, CrI = credible interval, FORM = formoterol, FEV_1 = forced expiratory volume in one second, FF = fluticasone furoate, FP = fluticasone propionate, MMF = mometasone furoate, SAL = salmeterol, VI = vilanterol

Supplementary Figure 1 Networks of study treatments, by outcome of interest (sensitivity analysis population)

A: change from baseline in morning PEF; B: annual rate of moderate/severe exacerbations

A

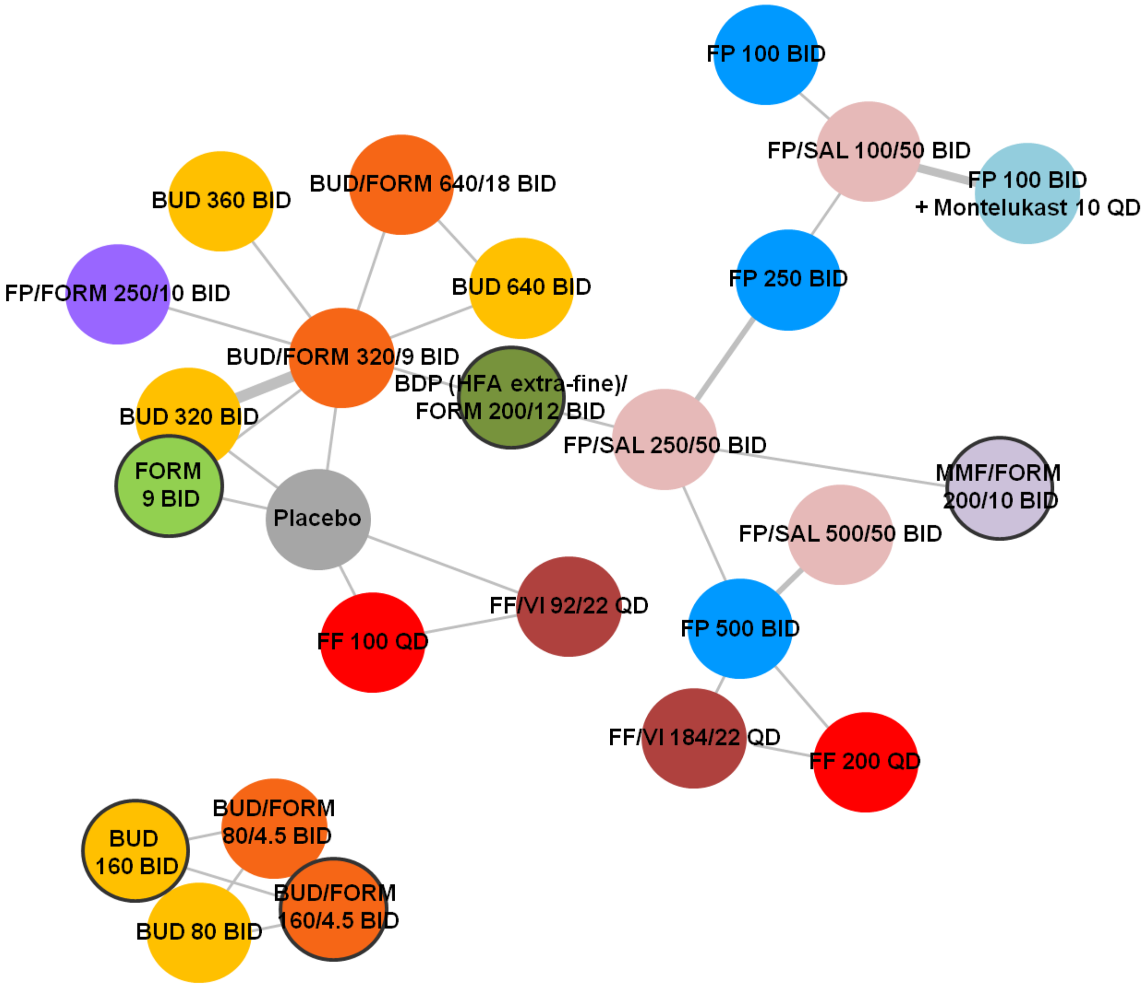

B

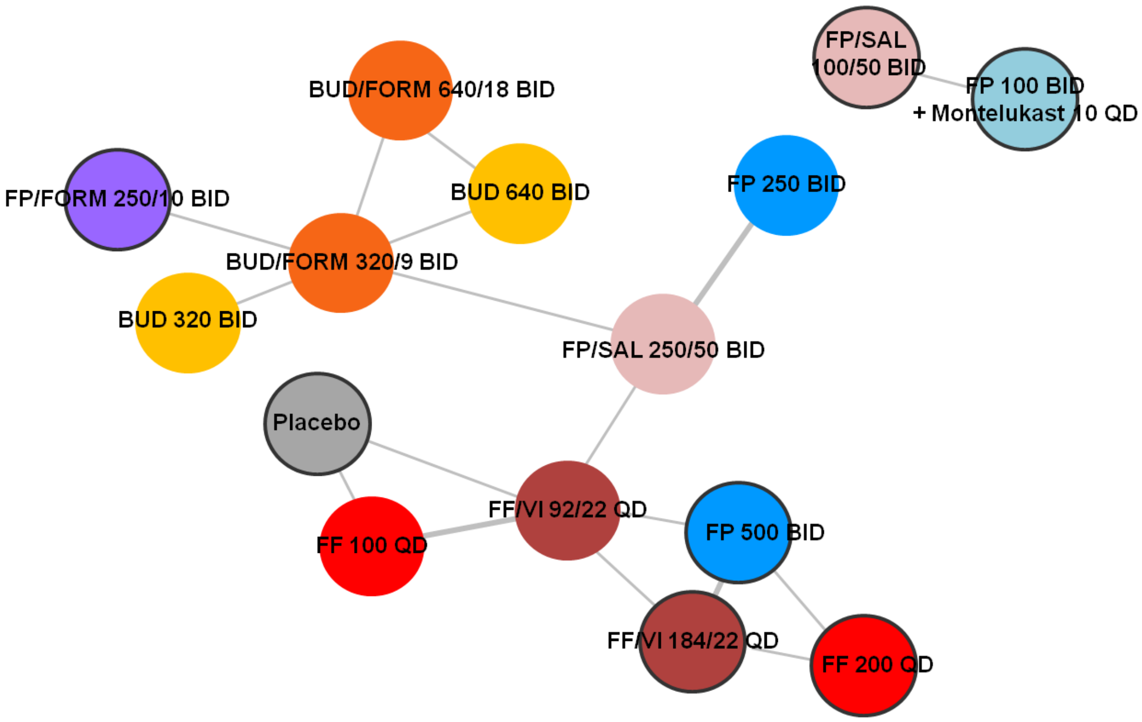

Note: All stated doses are µg. Delivered doses are given for FF/VI at the strengths licenced in Europe for the treatment of asthma, and for BUD/FORM. For all other treatments, nominal doses are given. Connecting lines represent studies included in the model that directly compare the two treatments. The thickness of the line is proportional to the number of studies comparing the two treatments. Treatments outlined in dark grey are included in the sensitivity analysis only (i.e. not in the primary analysis).

BDP = beclomethasone dipropionate, BID = twice daily, BUD = budesonide, FORM = formoterol, FEV_1 = forced expiratory volume in one second, FF = fluticasone furoate, FP = fluticasone propionate, HFA = hydrofluoroalkane, MMF = mometasone furoate, QD = once daily, SAL = salmeterol, VI = vilanterol

Data sources used in systematic literature search to identify shortlisted studies

**Data sources**	
**Databases**	Clinical Publication Databases<br>• Medline (OvidSP)<br>• Medline In-Process Citations & Daily Update (OvidSP)<br>• Embase (OvidSP)<br>• Cochrane Database Of Systematic Reviews (CDSR) (Wiley)<br>• Cochrane Central Register of Controlled Trials (CENTRAL) (Wiley)<br>• Database of Abstracts of Reviews of Effects (DARE) (Wiley)<br>• Health Technology Assessment Database (HTA) (Wiley)<br>• NIHR Health Technology Assessment Programme (Internet)<br>• PROSPERO (International Prospective Register of Systematic Reviews) (Internet) http://www.crd.york.ac.uk/prospero/<br><br>Clinical trials registers<br>• NIH Clinicaltrials.gov (Internet) http://www.clinicaltrials.gov/<br>• Current Controlled Trials (Internet) http://www.controlled-trials.com/<br>• WHO International Clinical Trials Registry Platform (ICTRP) (Internet) http://www.who.int/ictrp/en/<br>• European Medicines Agency European Public Assessment Reports (EMA EPARs) (http://www.ema.europa.eu/htms/human/epar/a.htm)<br>• FDA website<br>• Conference abstracts from COPD conferences (American Thoracic Society (ATS), European Respiratory Society (ERS), American College of Chest Physicians (ACCP), from 2010).

Study selection for primary analyses of outcomes of interest

Of the 97 studies identified by the systematic literature search, 36 were considered for inclusion in the four outcome analyses. The remaining 61 were excluded as follows:

- 10 were excluded because the study duration was ≤8 weeks
- 1 was excluded because patients aged <12 years were included
- 18 were excluded because patients were not on ICS or ICS/LABA at randomisation
- 18 were excluded because patients were not uncontrolled/symptomatic at baseline
- 14 were excluded because at least one of the treatment arms comprised a flexible dosing regimen and/or a dose-ranging arm.

Change from baseline PEF

- 18 of the 36 studies were excluded, all because they did not report usable change from baseline morning PEF data averaged over the whole study period. 18 studies were therefore included in the PEF MTC (Figure 1a).

Change from baseline FEV_1

- 8 of the 36 studies were excluded because they did not report usable FEV_1 data or did not report FEV_1 change from baseline. 28 studies were therefore included in the FEV_1 MTC (Figure 1b).

Annual rate of moderate/severe exacerbations

- 30 of the 36 studies were excluded from the exacerbation rates analysis:
    - 1 was not included in the analysis because it excluded patients who experienced ≥1 life-threatening exacerbation requiring hospitalisation in the prior 12 months
    - 20 studies were excluded because they did not report exacerbation data or did not report data in a form usable for the estimation of event rates

   - 3 were excluded because their exacerbation definitions were inconsistent with the ATS/ERS recommendations (Reddel, 2009), e.g. because they allowed symptom, rescue use or lung function changes alone to count as exacerbations
   - 6 were excluded because they withdrew patients once they experienced an exacerbation.
- 6 studies were therefore included in the exacerbations MTC (Figure 1c).

Change from baseline AQLQ score

- 29 of the 36 studies were excluded, all because they did not report AQLQ data as change from baseline. 7 studies were therefore included in the AQLQ MTC (Figure 1d).
