# Supplementary material for: Once-daily fluticasone furoate/vilanterol versus twice daily combination therapies in asthma–mixed treatment comparisons of clinical efficacy
Source: Asthma Res Pract. 2016 Feb 8;2:4. doi: 10.1186/s40733-015-0016-0 (PMC5142397; doi:10.1186/s40733-015-0016-0)
Supplement: Additional file 2: — Table S1: Summary of studies and treatment arms included in the updated primary mixed treatment comparison analysis. Table S2: Posterior probability of non-inferiority for FF/VI versus other relevant ICS/LABA combination therapies Table S3: Posterior probability of non-inferiority for FF/VI versus other relevant ICS/LABA combination therapies. (DOCX 22 kb) [file 40733_2015_16_MOESM2_ESM.docx]

**Supplement 1**

**Once-daily fluticasone furoate/vilanterol versus twice daily combination therapies in asthma – mixed treatment comparisons of clinical efficacy**

Henrik Svedsater^1^, Gillian Stynes^1^, Jaro Wex^1^*, Lucy Frith^2^, David Leather^2^, Emanuela Castelnuovo^3^*, Michelle Detry^4^, Scott Berry^4^

^1^Value Evidence and Outcomes, GlaxoSmithKline, Stockley Park, UK. ^2^Respiratory Medicines Development Centre, GlaxoSmithKline, Stockley Park, UK. ^3^Health Investment Evidence, Global Health Outcomes, GlaxoSmithKline, Stockley Park, UK. ^4^Berry Consultants LLC, Austin, TX, USA

*Formerly

The mixed treatment comparison was updated with more recently published data; studies published between 18th December 2012 and 30th July 2014 were identified by an update of the systematic review. Seven additional studies were identified for inclusion in this update, thus a total of 38 studies were included.

**Table S1** Summary of studies and treatment arms included in the updated primary mixed treatment comparison analysis

|  | **N (%)** |  | **N (%)** |
| --- | --- | --- | --- |
| **Total studies** | **38** | **Total treatment arms** | **100** |
| Endpoint reported |  | Placebo | 4 (4 %) |
| Change from baseline in FEV_1_ | 35 (92 %) | FF/VI 100/25 QD | 6 (6 %) |
| Change from baseline in PEF | 22 (58 %) | FF/VI 200/25 QD | 4 (4 %) |
| Annual rate of exacerbations | 7 (18 %) | FF 100 QD | 3 (3 %) |
| Change from baseline in AQLQ | 10 (26 %) | FF 200 QD | 1 (1 %) |
| Mean age reported | 42.09 | SFC 100/50 BID | 8 (8 %) |
| Mean percent male | 39.93 | SFC 250/50 BID | 11 (11 %) |
| Mean baseline FEV_1_ | 2.25 | SFC 500/50 BID | 5 (5 %) |
|  |  | BUD/FOR 320/9 BID | 13 (13 %) |
|  |  | BUD/FOR 640/18 BID | 1 (1 %) |
|  |  | BUD/FOR 160/4.5 BID | 1 (1 %) |
|  |  | BUD/FOR 80/4.5 BID | 3 (3 %) |
|  |  | BUD 320 BID | 5 (5 %) |
|  |  | BUD 640 BID | 2 (2 %) |
|  |  | BUD 360 BID | 1 (1 %) |
|  |  | BUD 160 BID | 1 (1 %) |
|  |  | BUD 80 BID | 2 (2 %) |
|  |  | BDP (HFA extra-fine) /FOR 200/12 BID | 2 (2 %) |
|  |  | FOR 9 BID | 1 (1 %) |
|  |  | FP 250 BID | 6 (6 %) |
|  |  | FP 500 BID | 6 (6 %) |
|  |  | FP 100 BID | 1 (1 %) |
|  |  | FP 100 BID + Montelukast 10 QD | 3 (3 %) |
|  |  | FP/FOR 250/10 BID | 3 (3 %) |
|  |  | MMF/FOR 200/10 BID | 2 (2 %) |
|  |  | MMF/FOR 400/10 BID | 1 (1 %) |
|  |  | BDP/FOR 100/6 BID + BDP/FOR PRN | 1 (1 %) |
|  |  | BDP/FOR 100/6 BID + SALBUTAMOL PRN | 1 (1 %) |
|  |  | FOR 10 BID | 1 (1 %) |
|  |  | FP/FOR 250/12 BID | 1 (1 %) |

*Note:* All stated doses are mcg. Delivered doses are given for FF/VI at the strengths licenced in Europe for the treatment of asthma, and for BUD/FORM. For all other treatments, nominal doses are given.

**Table S2** Posterior probability of non-inferiority for FF/VI versus other relevant ICS/LABA combination therapies*

*For studies requiring patients to be treated with ICS or ICS/LABA at baseline*; full covariate model.

*Other relevant ICS/LABA: FP/SAL 250/50 mcg and 500/50 mcg; BUD/FORM 320/9 mcg and 640/18 mcg

**a**) change from baseline in morning PEF. **b**) change from baseline in FEV_1_. **c**) annual rate of moderate/severe exacerbations^†^. **d**) AQLQ Total score.

**A**

| Treatment | Comparator | Mean difference, l  (95 % CrI) | Probability of non-inferiority  Margin (l/min) | |
| --- | --- | --- | --- | --- |
|  |  |  | **12** | **15** |
| FF/VI 92/22 | FP/SAL 250/50 | 13.97 (4.00–23.93) | >99 % | >99 % |
| FF/VI 92/22 | BUD/FORM 320/9 | 13.09 (3.49–22.70) | >99 % | >99 % |
| FF/VI 184/22 | FP/SAL 500/50 | 9.31 (1.26–17.36) | >99 % | >99 % |
| FF/VI 184/22 | BUD/FORM 640/18 | 10.41 (-1.59–22.40) | >99 % | >99 % |

**B**

| Treatment | Comparator | Mean difference, ml  (95 % CrI) | Probability of non-inferiority  Margin (ml) | | |
| --- | --- | --- | --- | --- | --- |
|  |  |  | **75** | **100** | **125** |
| FF/VI 92/22 | FP/SAL 250/50 | 0.00 (-0.05–0.05) | >99 % | >99 % | >99 % |
| FF/VI 92/22 | BUD/FORM 320/9 | 0.02 (-0.03–0.08) | >99 % | >99 % | >99 % |
| FF/VI 184/22 | FP/SAL 500/50 | 0.09 (0.02–0.16) | >99 % | >99 % | >99 % |
| FF/VI 184/22 | BUD/FORM 640/18 | 0.04 (-0.07–0.14) | 99 % | >99 % | >99 % |

**C**

| Treatment | Comparator | Rate ratio  (95 % CrI) | Probability of non-inferiority  Margin (event rate ratio) | |
| --- | --- | --- | --- | --- |
|  |  |  | **10 %** | **20 %** |
| FF/VI 92/22 | FP/SAL 250/50 | 0.91 (0.38–2.06) | 80 % | 84 % |
| FF/VI 92/22 | BUD/FORM 320/9 | 0.80 ( 0.30–1.64) | 88 % | 91 % |

^†^Only study length was included as a covariate in analysis of moderate/severe exacerbations data.

**D**

| Treatment | Comparator | Mean difference, units  (95 % CrI) | Probability of non-inferiority  Margin (units) | |
| --- | --- | --- | --- | --- |
|  |  |  | **0.25** | **0.5** |
| FF/VI 92/22 | FP/SAL 250/50 | 0.01 (-0.13–0.15) | >99 % | >99 % |
| FF/VI 92/22 | BUD/FORM 320/9 | -0.15 (-0.40–0.10) | 79 % | >99 % |

*Note:* All stated doses are mcg.

AQLQ = Asthma Quality of Life Questionnaire, BUD = budesonide, CrI = credible interval, FORM = formoterol, FEV_1_ = forced expiratory volume in 1 second, FF = fluticasone furoate, FP = fluticasone propionate, PEF = peak expiratory flow, SAL = salmeterol, VI = vilanterol

**Table S3** Posterior probability of non-inferiority for FF/VI versus other relevant ICS/LABA combination therapies*

*For studies requiring patients to be treated with ICS only at baseline.**Other relevant ICS/LABA: FP/SAL 250/50 mcg and 500/50 mcg; BUD/FORM 320/9 mcg and 640/18 mcg

**a**) change from baseline in morning PEF. **b**) change from baseline in FEV_1_. **c**) annual rate of moderate/severe exacerbations^†^. **d**) AQLQ Total score.

**A**

| Treatment | Comparator | Mean difference, l  (95 % CrI) | Probability of non-inferiority  Margin (l/min) | |
| --- | --- | --- | --- | --- |
|  |  |  | **12** | **15** |
| FF/VI 92/22 | FP/SAL 250/50 | 13.82 (3.57–24.06) | >99 % | >99 % |
| FF/VI 92/22 | BUD/FORM 320/9 | 12.75 (2.65–22.85) | >99 % | >99 % |
| FF/VI 184/22 | FP/SAL 500/50 | 9.53 (1.36–17.70) | >99 % | >99 % |
| FF/VI 184/22 | BUD/FORM 640/18 | 10.15 (-2.26–22.56) | >99 % | >99 % |

**B**

| Treatment | Comparator | Mean difference, ml  (95 % CrI) | Probability of non-inferiority  Margin (ml) | | |
| --- | --- | --- | --- | --- | --- |
|  |  |  | **75** | **100** | **125** |
| FF/VI 92/22 | FP/SAL 250/50 | 0.00 (-0.05–0.05) | >99 % | >99 % | >99 % |
| FF/VI 92/22 | BUD/FORM 320/9 | 0.02 (-0.03–0.08) | >99 % | >99 % | >99 % |
| FF/VI 184/22 | FP/SAL 500/50 | 0.10 (0.2–0.18) | >99 % | >99 % | >99 % |
| FF/VI 184/22 | BUD/FORM 640/18 | 0.03 (-0.07–0.13) | 98 % | 99 % | >99 % |

**C**

| Treatment | Comparator | Rate ratio  (95 % CrI) | Probability of non-inferiority  Margin (event rate ratio) | |
| --- | --- | --- | --- | --- |
|  |  |  | **10 %** | **20 %** |
| FF/VI 92/22 | FP/SAL 250/50 | 1.16 (0.42–3.33) | 74 % | 78 % |
| FF/VI 92/22 | BUD/FORM 320/9 | 0.99 (0.33–2.57) | 82 % | 86 % |

^†^Only study length was included as a covariate in analysis of moderate/severe exacerbations data.

**D**

| Treatment | Comparator | Mean difference, units  (95 % CrI) | Probability of non-inferiority  Margin (units) | |
| --- | --- | --- | --- | --- |
|  |  |  | **0.25** | **0.5** |
| FF/VI 92/22 | FP/SAL 250/50 | 0.05 (-0.09–0.20) | >99 % | >99 % |
| FF/VI 92/22 | BUD/FORM 320/9 | -0.15 (-0.37–0.08) | 82 % | >99 % |

*Note:* All stated doses are mcg.

AQLQ = Asthma Quality of Life Questionnaire, BUD = budesonide, CrI = credible interval, FORM = formoterol, FEV_1_ = forced expiratory volume in 1 second, FF = fluticasone furoate, FP = fluticasone propionate, SAL = salmeterol, VI = vilanterol
